# Cry1C rice doesn’t affect the ecological fitness of rice brown planthopper, *Nilaparvata lugens* either under RDV stress or not

**DOI:** 10.1038/s41598-020-73465-7

**Published:** 2020-10-02

**Authors:** Xuefei Chang, Linlin Sun, Duo Ning, Cong Dang, Hongwei Yao, Qi Fang, Yufa Peng, Fang Wang, Gongyin Ye

**Affiliations:** 1 State Key Laboratory of Rice Biology & Ministry of Agriculture and Rural Affairs Key Laboratory of Molecular Biology of Crop Pathogens and Insects, Institute of Insect Sciences, Zhejiang University, Hangzhou, 310058 China; 2 State Key Laboratory for Biology of Plant Diseases and Insect Pests, Institute of Plant Protection, Chinese Academy of Agricultural Sciences, Beijing, 100193 China

**Keywords:** Ecology, Agroecology

## Abstract

The potential risks of *Bt* rice on non-target arthropods (NTAs) should be evaluated and defined before commercial production. Recently, effects of *Bt* rice on NTAs under abiotic and biotic stress conditions attracted much attention. Here we reported the effects of *Bt* rice T1C-19 (Cry1C rice) on the non-target herbivore, *Nilaparvata lugens* (rice brown planthopper, BPH) with or without RDV (*rice dwarf virus*) infection conditions. BPH showed no feeding and oviposition preference between *Bt* rice T1C-19 and its non-*Bt* parental rice Minghui 63 (MH63), as well as between RDV-infected and RDV-free rice plants. Meanwhile, rice type, RDV infection status, and their interaction had little impacts on the survival, development and fecundity of BPH. By comparison with non-*Bt* control, *Bt* rice T1C-19 with or without RDV infection had no significant effects on the life-table parameters of BPH including *r*_*m*_, *R*_*0*_, *T*, DT and *λ*. Thus, it could be concluded that *Bt* rice T1C-19 doesn’t affect the ecological fitness of BPH either under RDV stress or not.

## Introduction

Rice (*Oryza sativa* L.) is one of the most important food crops, which fed more than half of the world’s population^1^. However, rice production is usually negatively affected by insect pests, leading to great economic loss and threatening food security^2,3^. Developing insect-resistant rice varieties is an economically-effective method for insect pests controlling. Since the early 1990s, a series of genetically modified rice events expressing Cry proteins derived from the soil bacterium *Bacillus thuringiensis* (*Bt*) have been created. They were proved to have high efficiency in controlling lepidopteran pests such as rice stem borers *Chilo suppressalis* (Lepidoptera: Crambidae), *Scirpophaga incertulas* (Lepidoptera: Crambidae), *Sesamia inferens* (Lepidoptera: Noctuidae) and rice leaf folder, *Cnaphalocrocis medinalis* (Lepidoptera: Pyralidae)^1,4^. Two Cry1Ab/Cry1Ac rice lines (Huahui 1 and *Bt-*Shanyou 63) had been issued biosafety certificates in China^1^. However, they have not been realized for releasing to farmers’ growing, which might be due to public consideration on agro-ecological and food security.

One of the main concerns on ecological risk is the effects of *Bt* rice on non-target arthropods (NTAs). A range of studies have been done with different *Bt* rice lines on various NTAs. For most cases, no significant impacts were found on NTAs. Zero effect of *Bt* rice lines including TT51 (Cry1Ab/Cry1Ac rice), T1C-19 (Cry1C rice) and T2A-1 (Cry2A rice) was found on the survival, development and fecundity of rice brown planthopper (BPH) *Nilaparvata lugens* (Hemiptera: Delphacidae), and also on physiological processes of digestion, detoxification and immune responses of BPH^5^. And no significant difference was detected on biological parameters of parasitoids *Pseudogonatopus flavifemur* (Hymenoptera: Dryinidae) and *Anagrus nilaparvatae* (Hymenoptera: Mymaridae) when nymph or honeydew of BPH reared with T1C-19 and T2A-1 were used as food^6,7^. It was reported that *Bt*-Shanyou 63 (Cry1Ab/Cry1Ac rice) did not differ in the quality from its non-*Bt* parental rice Shanyou 63, and would be a safe food source for *Daphnia magna* (Cladocera: Daphniidae), a model organism which had been extensively used in ecotoxicology studies^8^. Negative impacts of KMD2 (Cry1Ab rice) on the fecundity of non-target herbivores BPH and rice thrips *Stenchaetothrips biformis* (Thysanoptera: Thripidae) were detected, which indicated that KMD2 would not stimulate the outbreak of these two non-target pests^9,10^. However, positive effects on green rice leafhoppers (GRLHs) *Nephotettix cincticeps* (Hemiptera: Cicadellidae) were found when fed on KMD1 (Cry1Ab rice) and T2A-1. Enhanced fecundity was found on GRLH fed on KMD1 compared with its non-*Bt* parental rice Xiushui 11^11^. And the survival rate of GRLH nymphs was increased when feeding on T2A-1 rice plants, compared with its non-*Bt* parental rice Minghui 63 (MH63)^12^. These results indicated that the effects of *Bt* rice on NTAs depending on *Bt* rice events and also NTA species tested, thus should be assessed case by case.

Recently, effects of *Bt* crops on indirect multi-trophic interactions received much attention from researchers as complicated interactions are common in real environment. It was reported that reduced level of *Bt*-sensitive lepidopteran caterpillars-induced terpenoids in *Bt* cotton benefited the non-target herbivore *Aphis gossypii* (Hemiptera: Aphididae)^13^. Changes in plant terpenoid levels as a response to *Heliothis virescens* damage might also influence the host quality of aphids for the parasitoid *Lysiphlebus testaceipes* (Hymenoptera: Aphidiidae). Fewer mummies of *L. testaceipes* were formed on *H. virescens* infested non-*Bt* cotton than on *Bt* cotton^14^. While caterpillar infested *Bt* rice attracted less BPH due to a relative lower level of plant volatiles released^15^. Plant pathogens including bacteria, fungi, virus would also interact with plant internal metabolism, impair plant growth and reproduction, and thus influence the behavior and performance of vector or non-vector insects. The non-vector insect *Bemisia tabaci* preferred to settle and oviposit on *Tomato spotted wilt virus* (TSWV)-infected *Datura stramonium* due to the alteration of plant volatiles^16^. *Rice black streak dwarf virus* (RBSDV) infection induced the increment of amino acids and soluble sugar in rice plants, and these changed metabolites improved the ecological fitness of the non-vector BPH^17^. There were also a few reports on combined effects of *Bt* rice and pathogen attacks on vector or non-vector insects. *Southern rice black-streaked dwarf virus* (SRBSDV)-infected T1C-19 or T2A-1 had little effects on the ecological adaptability of the non-vector insect BPH^18^. Main biological parameters of vector insect GRLHs such as egg hatching rate, nymph survival and female fecundity were also not affected by RDV (*rice dwarf virus*)-infected T1C-19 and T2A-1^19^.

*Rice dwarf virus* (RDV), a member of the genus *Phytoreovirus* in the family *Reoviridae*, is mainly transmitted by GRLHs in a persistent-propagative manner^19^. GRLHs acquire the virus by feeding on RDV-infected rice plants for a few minutes to several days. RDV could proliferates in the nearly 70% of GRLH individuals, which became viruliferous after a latent period of 2–3 weeks^20^. Viruliferous GRLHs can transmit RDV to rice plants throughout the lifetime. Infection with RDV would cause significant phenotype changes in rice plants such as dwarfism, white chlorotic specks on leaves, and incomplete panicle exsertion^21–23^. Here, the combined influences of *Bt* rice and RDV infection on an important insect BPH were investigated, which can transmit plant viruses such as *rice ragged stunt virus* (RRSV) and *rice grassy stunt virus* (RGSV), but not RDV^22,24–26^. The feeding and oviposition preference of BPH were investigated between *Bt* rice T1C-19 and its non-*Bt* parent rice MH63, as well as those between RDV-infected and RDV-free rice plants. Life-table parameters were used to evaluate the ecological fitness of BPH on both RDV-infected and RDV-free T1C-19 or MH63 rice plants. The present study would fulfill the multi-factor interactions between *Bt* rice and other biotic factors on NTAs, which can help to predict the sustainability of *Bt* rice in the system of rice pest integrated management.

## Results

### Feeding and oviposition preference of BPH

**Figure 1:**
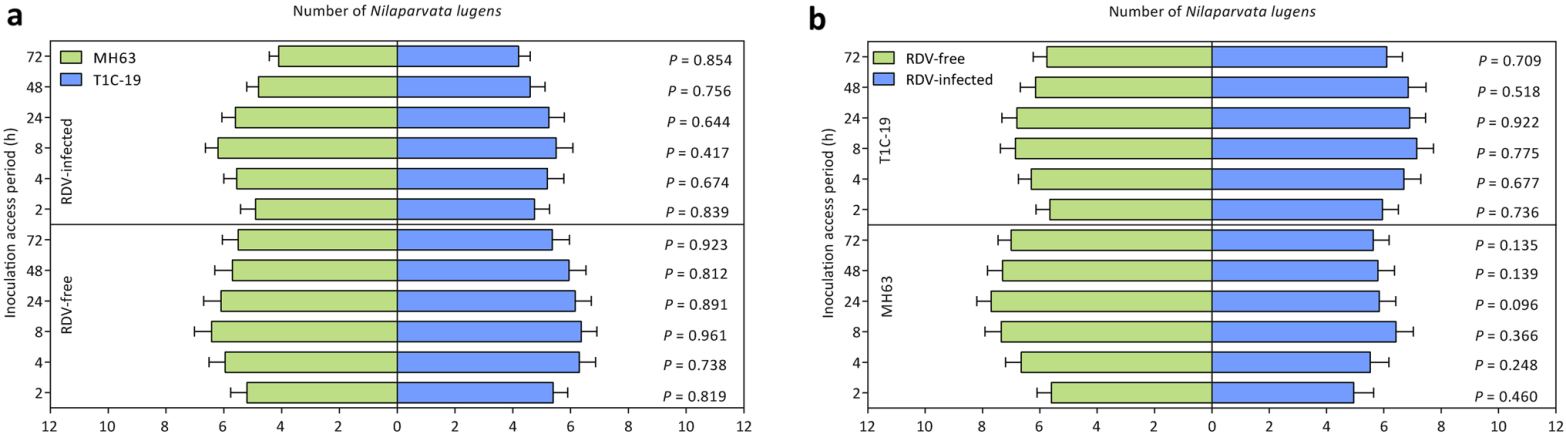
The effects of rice type (**a**) and RDV infection (**b**) on *Nilaparvata lugens* feeding preference. All choice tests were performed with 20 replicates, each consisting of 15 gravid female adults. Values are mean ± standard error. Data of each test were analyzed by paired-sample *t*-test (*P* > 0.05 indicated no significant difference).

**Figure 2:**
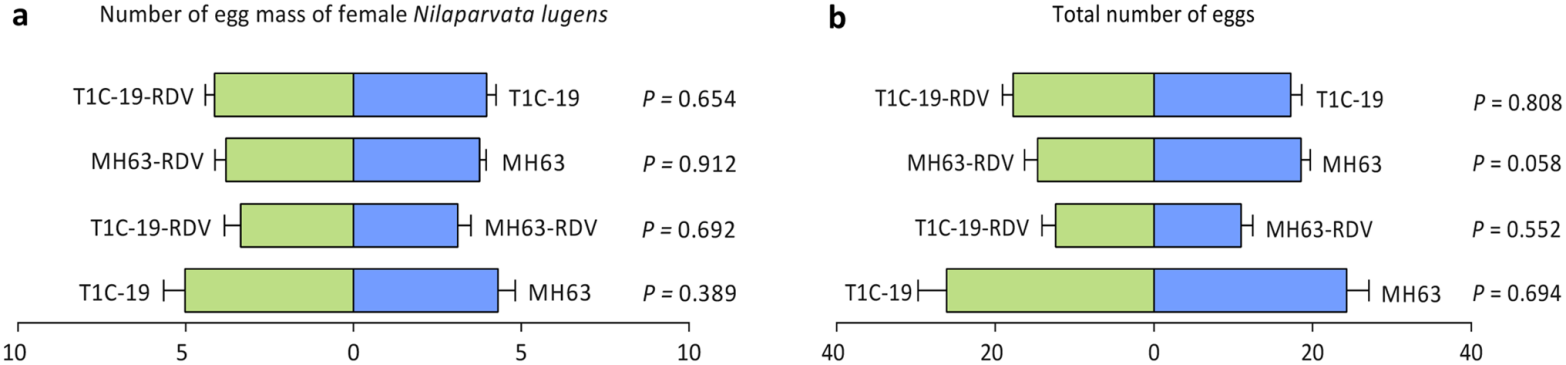
The oviposition preference of *N. lugens*. (**a**) Number of egg mass laid by *N. lugens*. (**b**) Total number of eggs laid by *N. lugens*. All choice tests were performed with 20 replicates, each consisting of 15 gravid female adults. Values are mean ± standard error. Data of each test were analyzed by paired-sample *t*-test (*P* > 0.05 indicated no significant difference).

Feeding and oviposition preference of BPH were investigated in homemade plastic cages in pairs independently (MH63 vs T1C-19, MH63-RDV vs T1C-19-RDV, MH63 vs MH63-RDV, T1C-19 vs T1C-19-RDV). For feeding preference, the number of BPH female adults settled on each rice type was calculated within 72 h post-inoculation (PI) in each test. Number of BPH on T1C-19 did not differ from that on MH63 either under RDV-free or RDV infection status (Fig. 1a, *P* > 0.05). Meanwhile, number of BPH on RDV-infected T1C-19 or MH63 showed no significant difference with that on RDV-free rice plants either (Fig. 1b, *P*  > 0.05). Thus, rice type and RDV infection status had no impact on BPH feeding preference within 72 h PI. For oviposition preference, the number of egg mass and total number of eggs per female adult were counted on each rice plants at 72 h PI. No significant difference was seen in the two parameters between BPH on different rice types both under RDV-free and RDV infection status (Fig. 2, *P* > 0.05). Similarly, the fecundity of BPH on RDV-infected rice plants also had no significant difference with those on RDV-free rice plants (Fig. 2, *P* > 0.05). Therefore, rice type and RDV infection had no effects on BPH oviposition preference.

### Combined effects of *Bt* rice and RDV infection on biological parameters of BPH

#### Survival, development and fecundity of BPH

**Figure 3:**
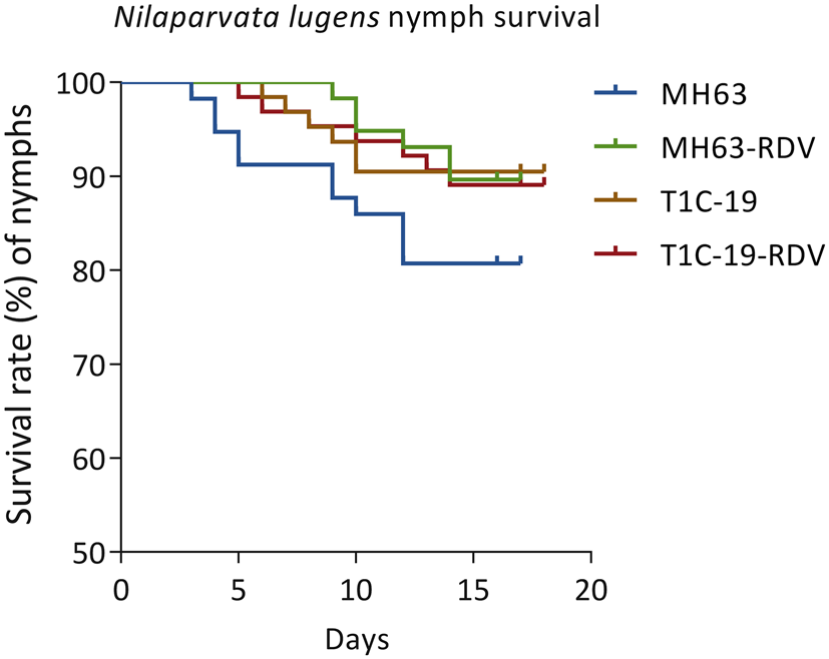
The survivorship curves of *N. lugens* over a 20-day period when fed on MH63, MH63-RDV, T1C-19, T1C-19-RDV rice plants. *n* = 64 for each test.

**Figure 4:**
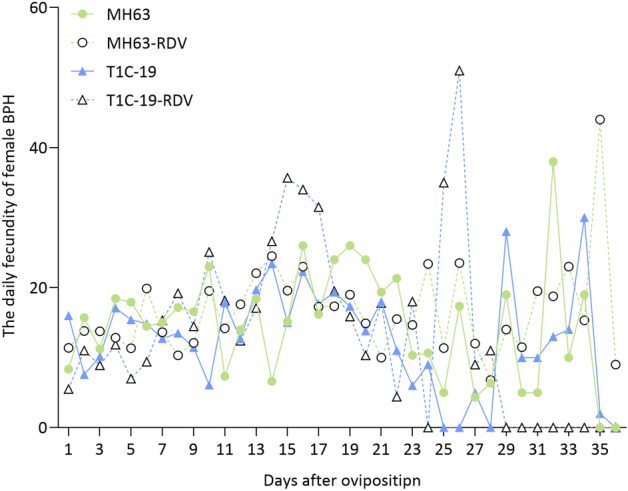
The daily fecundity of *N. lugens* females fed on MH63, MH63-RDV, T1C-19, T1C-19-RDV rice plants. *n* = 14, 19, 15, 15 for MH63, MH63-RDV, T1C-19, T1C-19-RDV respectively. The effects of rice type, RDV infection and their interaction on the daily fecundity of female BPH were analyzed using a mixed-model, repeated-measures analysis of variance (ANOVA).

The survivorship curves of BPH nymphs showed no significant difference among the four treatments (Fig. 3, χ^2^ = 5.734; *df* = 3; *P* = 0.125). Rice type, RDV infection, and their interaction (rice type × RDV infection status) had no impact on BPH survival rate (rice type, χ^2^ = 1.519; *df* = 1; *P* = 0.218; RDV infection, χ^2^ = 1.332; *df* = 1; *P* = 0.249; rice type × RDV infection, χ^2^ = 5.300; *df* = 3; *P* = 0.151). Biological parameters including egg hatching rate and egg duration, nymph duration, adult longevity and body weight, as well as female fecundity were listed in Table 1. Rice type, RDV infection status and their interaction had no significant effects on egg hatching rate, adult longevity and body weight, and also the pre-oviposition period, oviposition period and fecundity (total number of eggs laid per female) of female adults (Table 1, *P* > 0.05). The egg duration was significantly affected by rice type (*P* < 0.001), and the interaction of rice type and RDV infection (*P* = 0.001), but not by RDV infection status (*P* > 0.05). Egg duration of BPH was significantly longer on T1C-19-RDV rice plants than on other rice plants (T1C-19, MH63, MH63-RDV) (Table 1). Male nymph duration was significantly affected by rice type (*P* = 0.018), RDV infection status (*P* = 0.017) and their interaction (*P* = 0.013). But female nymph duration was only affected by rice type (*P* = 0.005). Nymph duration on T1C-19 was significantly longer than that on MH63 under RDV-free condition, but not RDV infection status (Table 1). Rice type, RDV infection and their interaction had no significant impacts on the daily fecundity of BPH either (Fig. 4, rice type, *F* = 0.861, *df* = 1, *P* = 0.358; RDV infection, *F* = 2.751, *df* = 1, *P* = 0.103; rice type × RDV infection, *F* = 0.013, *df* = 1, *P* = 0.910).

**Table 1:** Effects of rice type, RDV infection status and interaction of rice type and RDV infection status on biological parameters of *N. lugens* under laboratory conditions.

Parameters	MH63	MH63-RDV	T1C-19	T1C-19-RDV	Two-way ANOVA
Eggs hatching rate (%)	85.22 ± 8.54a (12)	75.47 ± 6.97a (18)	83.46 ± 7.91a (14)	80.99 ± 8.54a (12)	*F*_*A*_ = 0.06, *P* = 0.815; *F*_*B*_ = 0.58, *P* = 0.449; *F*_*A*×*B*_ = 0.21, *P* = 0.652
Egg duration (days)	8.77 ± 0.09b (166)	8.52 ± 0.09b (169)	8.79 ± 0.08b (191)	9.15 ± 0.10a (137)	*F*_*A*_ = 13.76, *P* < 0.001; *F*_*B*_ = 0.40, *P* = 0.527; *F*_*A*×*B*_ = 11.69, *P* = 0.001
Female nymph duration (days)	14.22 ± 0.23b (23)	14.57 ± 0.21ab (27)	15.05 ± 0.18a (37)	14.89 ± 0.21ab (26)	*F*_*A*_ = 8.36, *P* = 0.005; *F*_*B*_ = 0.10, *P* = 0.752; *F*_*A*×*B*_ = 1.28, *P* = 0.260
Male nymph duration (days)	12.71 ± 0.23b (21)	13.79 ± 0.19a (24)	13.85 ± 0.23a (19)	13.77 ± 0.19ab (30)	*F*_*A*_ = 5.85, *P* = 0.018; *F*_*B*_ = 5.90, *P* = 0.017; *F*_*A*×*B*_ = 6.42, *P* = 0.013
Body weight of female adults (mg/adult)	2.32 ± 0.14a (10)	2.23 ± 0.12a (13)	2.18 ± 0.13a (11)	2.35 ± 0.13a (12)	*F*_*A*_ = 0.01, *P* = 0.941; *F*_*B*_ = 0.09, *P* = 0.760; *F*_*A*×*B*_ = 1.02, *P* = 0.319
Body weight of male adults (mg/adult)	1.28 ± 0.04a (10)	1.31 ± 0.03a (11)	1.33 ± 0.03a (12)	1.40 ± 0.04a (10)	*F*_*A*_ = 3.90, *P* = 0.055; *F*_*B*_ = 2.29, *P* = 0.139; *F*_*A*×*B*_ = 0.41, *P* = 0.527
Female longevity (days)	16.59 ± 1.91a (17)	22.18 ± 1.68a (22)	17.50 ± 1.44a (30)	17.58 ± 1.81a (19)	*F*_*A*_ = 1.16, *P* = 0.286; *F*_*B*_ = 2.72, *P* = 0.102; *F*_*A*×*B*_ = 2.58, *P* = 0.112
Male longevity (days)	21.57 ± 2.04a (14)	18.83 ± 1.80a (18)	18.75 ± 1.91a (16)	20.26 ± 1.59a (23)	*F*_*A*_ = 0.14, *P* = 0.707; *F*_*B*_ = 0.11, *P* = 0.740; *F*_*A*×*B*_ = 1.33, *P* = 0.253
Pre-oviposition period (days)	2.50 ± 0.23a (18)	2.35 ± 0.22a (20)	2.25 ± 0.25a (16)	2.47 ± 0.26a (15)	*F*_*A*_ = 0.08, *P* = 0.782; *F*_*B*_ = 0.02, *P* = 0.890; *F*_*A*×*B*_ = 0.58, *P* = 0.448
Oviposition period (days)	15.21 ± 2.27a (14)	19.32 ± 1.95a (19)	13.27 ± 2.20a (15)	14.33 ± 2.20a (15)	*F*_*A*_ = 2.58, *P* = 0.114; *F*_*B*_ = 1.43, *P* = 0.236; *F*_*A*×*B*_ = 0.50, *P* = 0.485
Fecundity (eggs per female)	232.64 ± 51.15a (14)	327.26 ± 43.91a (19)	198.67 ± 49.41a (15)	262.40 ± 49.41a (15)	*F*_*A*_ = 1.04, *P* = 0.313; *F*_*B*_ = 2.66, *P* = 0.108; *F*_*A*×*B*_ = 0.10, *P* = 0.752

The data are presented as means ± standard error. Number of replicates is indicated in parentheses. Biological parameters were analyzed using general linear models (GLMs) followed by Tukey’s multiple range test. Different letters in the same row indicate significant difference among treatments (*P* < 0.05). Factor A: rice type; Factor B: RDV infection status; F_A×B_: the interaction of rice type and RDV infection status.

#### Life-table parameters of BPH

Life-table parameters of BPH including the intrinsic rate of natural increase (*r*_*m*_), the net reproductive rate (*R*_*0*_), the mean generation time (*T*), the doubling time (DT) and the finite capacity of increase (λ) were not affected by rice type or RDV infection status (Table 2, Supplementary Table S1, *P* > 0.05).

**Table 2:** Life table parameters of *N. lugens* feeding on MH63, MH63-RDV, T1C-19, T1C-19-RDV under laboratory conditions^a^.

Parameters^b^	MH63	MH63-RDV	T1C-19	T1C-19-RDV
*r*_*m*_	0.20 ± 0.0025a	0.20 ± 0.0011a	0.21 ± 0.0020a	0.20 ± 0.0016a
*R*_*0*_	92.67 ± 4.76a	151.96 ± 5.90a	119.18 ± 6.25a	106.29 ± 4.71a
*T* (days)	22.56 ± 0.37a	25.33 ± 0.24a	22.79 ± 0.26a	23.36 ± 0.16a
DT (days)	3.44 ± 0.04a	3.48 ± 0.02a	3.29 ± 0.03a	3.46 ± 0.03a
*λ*	1.22 ± 0.0030a	1.22 ± 0.0013a	1.23 ± 0.0025a	1.22 ± 0.0020a

^a^All life-table parameters were calculated using an SAS program written by Maia et al.^41^ using the jackknife method. Means ± standard error (SE) in the same rows followed by the same letters are not significantly different at *P* < 0.05 level.

^b^*r*_*m*_: the intrinsic rate of natural increase, *R*_*0*_: the net reproductive rate; *T*: the mean generation time; DT: the doubling time; *λ*: the finite capacity of increase.

## Discussion

The effects of *Bt* rice on NTAs including herbivores, predators, parasitoids and detritivores have been studied thoroughly in past 20 years^27^. Recently, much attention has been paid to effects of *Bt* crops on indirect multi-trophic interactions, whereas few attentions were paid to the combined effects of *Bt* rice with other biotic or abiotic factors^13–15,18,19,28–31^. Interactions between plants and pathogens on insects are complex. In field system, vector and non-vector insects usually shared the same host plants and compete for plant resources^32^. It was reported that RDV-infected rice plants were more attractive to non-viruliferous vector insect GRLHs for feeding^33^. However, no such effect was found on the non-vector insect BPH in the current study. Meanwhile, BPH showed no feeding and oviposition preference between *Bt* rice T1C-19 and its non-*Bt* parental rice MH63 under RDV-free or RDV infection status (Fig. 1a), which is similar to the previous study on GRLHs^19^. In general, our results indicated that rice type and RDV infection status had no effect on feeding and oviposition preference of BPH (Figs. 1, 2). Consistently, BPH showed no feeding preference between undamaged Huahui 1 (Cry1Ab/Cry1Ac rice) and its non-*Bt* parental rice MH63 in both laboratory and field-cage experiments^15^. And rough rice grains of T1C-19 did not change the host preference of the non-target stored-product pest, *Rhyzopertha dominica* (Coleoptera: Bostrichidae) and its parasitoid wasp *Anisopteromalus calandrae* (Hymenoptera: Pteromalidae)^34^.

The effects of rice type, RDV infection status and their interaction on biological parameters of BPH were also investigated. Although difference in some biological parameters were found between BPH fed on *Bt* rice T1C-19 and its non-*Bt* parental rice MH63, the survival, female fecundity and life-table parameters of BPH including *r*_*m*_, *R*_*0*_, *T*, DT and *λ* showed no significant difference between T1C-19 and MH63 rice plants (Fig. 3; Tables 1, 2). It indicated that T1C-19 had no effect on population growth of BPH, which was in consistent with previous study^35^. RDV infection status also showed no impacts on BPH survival, development and female fecundity except for male nymph duration, although it significantly changed the phenotypes of rice plants. As a result, life-table parameters showed no significant changes between BPH fed on rice plants with or without RDV infection (Tables 1, 2). No significant effects of interaction between rice type and RDV infection status were found on BPH survival, development and female fecundity except for egg duration and male nymph duration. Based on our results, we concluded that the ecological fitness of BPH was not affected by rice type, RDV infection status and their interaction, as important biological parameters such as egg hatching rate, nymph survival rate and female fecundity showed no significant changes. Similarly, T1C-19 and T2A-1 infected with SRBSDV had no effect on survival, female fecundity and egg hatchability of the non-vector insect BPH^18^. However, there were also reports indicated that biological parameters of non-vector insects would be affected by virus infection. Ecological fitness of non-vector BPH and white-backed rice planthopper (WBPH) *Sogatella furcifera* (Hemiptera: Delphacidae) were improved on rice plants infected with *rice black streak dwarf virus* (RBSDV), which is transmitted by small BPH, *Laodelphax striatellus* (Hemiptera: Delphacidae)^17,36^. The nymph survival rate and female longevity of the non-vector insect BPH were significantly decreased when fed on SRBSDV-infected Y-liangyou1 rice plants^37^. Therefore, the impacts on biological parameters of vector or non-vector insects depend on the interactions between rice plants and viruses. Different viruses, or different rice lines, would result in different impacts.

In summary, our study showed that BPH had no feeding and oviposition preference between *Bt* rice T1C-19 and its non-*Bt* parental rice MH63 under RDV-free or RDV infection status. Similarly, *Bt* rice T1C-19 with or without RDV infection had negligible impact on biological parameters and life-table parameters of BPH at most cases, compared with the non-*Bt* control rice MH63. Thus, it is clear that *Bt* rice T1C-19 doesn’t affect the ecological fitness of BPH either under RDV stress or not. Moreover, life-table parameters are recommended to be used in the assessment of the effects of *Bt* crops on NTAs, as it can provide a comprehensive view of the biological characteristics of a given population under defined environmental conditions^38^. It would give us a more intuitive conclusion of the effects on the population growth, even when significant difference exists in some biological parameters.

## Materials and methods

### Insects

BPH and GRLH colonies were first collected from the paddy fields at the experiment farm of Zhejiang University, Hangzhou, China and then reared continuously on the susceptible rice ‘Taichung Native1′ (TN1) seedlings in 80-mesh nylon cage (50 cm length × 50 cm width × 50 cm height) in a climate chamber at 26 ± 1 °C, 75 ± 5% relative humidity and a photoperiod of 14 L:10 D. RDV acquisition of GRLHs was conducted as described by Wang et al.^19^. The 2nd or 3rd instar GRLH nymphs were fed with RDV-infected TN1 rice plants (preserved in our laboratory) after 2 h’ starving. These nymphs were transferred to RDV-free TN1 plants 48 h later to go through the latent period. Then the GRLH nymphs were individually released to RDV-free TN1 seedlings in plastic tubes (diameter 2.5 cm, height 25 cm, one seedling in each tube) and labeled. Seedlings were replaced every 2 days. All these seedlings were collected and transplanted into greenhouse, marked with number corresponding to GRLH nymphs individually. Viruliferous GRLHs were selected as RDV symptoms appear on the relative rice seedlings and collected approximately 15 days later^19^. These viruliferous GRLHs were then kept on the RDV-infected TN1 plants under the same conditions as described above until experiment.

### Plants

*Bt* rice line T1C-19 (Cry1C rice) and its non-*Bt* parental rice Minghui 63 (MH63) were used in this study^39^. To obtain RDV-infected rice plants, 4th to 5th instar viruliferous GRLH nymphs were inoculated on rice seedlings of 15 days old after 2 h’ starving. One GRLH nymph was inoculated on each seedling. For consistency, RDV-free plants were treated with non-viruliferous GRLHs similarly. After 48 h, all the GRLHs were removed and rice seedlings were transplanted in greenhouse hydroponically under natural lighting at the temperature of 25 ± 2 °C. Approximately 15 days later, only those showed RDV symptoms were collected and used as RDV-infected rice plants.

### Feeding and oviposition preference of BPH

Feeding and oviposition preference experiments were conducted using plastic pots (diameter 20 cm) and foam board with two holes spacing 10 cm apart. One seedling of 45-day-old was transplanted into each hole and cultured in Kimura B nutrient solution. The plots were covered by a transparent polyethylene-plastic cylinder (diameter 18 cm, height 50 cm) with two side-windows of nylon mesh in the middle. The top of cylinder was also covered by nylon mesh for ventilation^19^. Fifteen gravid female adults of BPH were transferred into each cage after 2 h’ starving. The numbers of BPH settled on each rice plant were recorded at 2, 4, 8, 24, 48, and 72 h post-inoculation (PI). The test groups were MH63 vs T1C-19, MH63-RDV vs T1C-19-RDV, MH63-RDV vs MH63, T1C-19-RDV vs T1C-19. Twenty separate replications were carried out for each test group. For oviposition preference, the number of egg mass of female BPH and the total number of eggs in each rice plant were investigated at 72 h PI.

### The effects of RDV-infected rice plants on biological parameters of BPH

RDV-free or RDV-infected rice seedlings of 45 days old were cleaned with tap water and transferred into glass tubes (diameter 3 cm, height 25 cm) with 20 ml Kimura B nutrient solution. One seedling was transplanted in each tube and renewed with the same rice type weekly till the end of experiment. Newly hatched BPH nymphs (within 12 h) were prepared as Lu et al.^35^ and inoculated on rice seedlings in glass tubes individually. The survival and developmental duration of BPH nymphs were recorded daily until adult emergence. There were 64 replications for each rice type. Once the adult BPH emerged, approximately 10 female and male adults were weighed on a microelectronic balance (AB135-S, Mettler Toledo, Switzerland, the accuracy of 0.01 mg). At the same time, female and male adults from the same treatment were mated, and a pair of BPH adults was transferred to a new tube with the corresponding rice plant. In total, there were 18, 20, 16, 15 pairs of BPH from MH63, MH63-RDV, T1C-19, T1C-19-RDV rice respectively. Rice plants were replaced daily until female BPH died. The adult longevity, pre-oviposition period and oviposition period of female adults were investigated. Once the male adult died, a new male adult would be supplemented until the female died. The numbers of eggs were counted by dissecting rice plants under a microscope. For development duration and hatching rate of eggs, eggs on the same day were collected, and the numbers of eggs hatched were recorded daily. Unhatched eggs were counted till no nymphs were observed.

### Statistical analysis

Analysis of data on feeding and oviposition preference, biological parameters, the survivorship curves of BPH nymphs and the daily fecundity of female BPH were performed using SPSS 20.0 software. The impacts of rice type or RDV infection on feeding and oviposition preference of BPH were analyzed by paired-sample *t*-test. Rice type and RDV infection on the survivorship curves of BPH nymphs were analyzed using the Kaplan–Meier method with Wilcoxon test. Interaction of rice type and RDV infection on survival rate of BPH nymphs were determined using Cox multivariate model. Data on biological parameters including development duration of nymphs, adult longevity and body weight, pre-oviposition period, oviposition period and female fecundity of BPH were analyzed using general linear models (GLMs) followed by Tukey’s multiple range test. The daily fecundity of female BPH was analyzed using a mixed-model, repeated-measures analysis of variance (ANOVA)^35^. The life-table parameters, including the intrinsic rate of natural increase (*r*_*m*_), the net reproductive rate (*R*_*0*_), the mean generation time (*T*), the doubling time (DT) and the finite capacity of increase (λ) were calculated and analyzed using SAS software (version 9.1) based on the Jackknife program^40,41^.

## Supplementary information

Supplementary information
