# Supplementary material for: Cry1C rice doesn’t affect the ecological fitness of rice brown planthopper, Nilaparvata lugens either under RDV stress or not
Source: Sci Rep. 2020 Oct 2;10:16423. doi: 10.1038/s41598-020-73465-7 (PMC7532440; doi:10.1038/s41598-020-73465-7)
Supplement: Supplementary file 1 — Supplementary information [file 41598_2020_73465_MOESM1_ESM.pdf]

Cry1C rice doesn't affect the ecological fitness of rice brown planthopper, *Nilaparvata lugens* either under RDV stress or not

Xuefei Chang[1], Linlin Sun[1], Duo Ning[1], Cong Dang[1], Hongwei, Yao[1], Qi Fang[1], Yufa Peng[2], Fang Wang[1]* & Gongyin Ye[1]*

[1]State Key Laboratory of Rice Biology & Ministry of Agriculture and Rural Affairs Key Laboratory of Molecular Biology of Crop Diseases and Insects, Institute of Insect Sciences, Zhejiang University, Hangzhou 310058, China

[2]State Key Laboratory for Biology of Plant Diseases and Insect Pests, Institute of Plant Protection, Chinese Academy of Agricultural Sciences, Beijing 100193, China

*Correspondence authors: Gongyin Ye, email: chu@zju.edu.cn; Fang Wang, email: wangf121@ zju.edu.cn

Table S1. The analysis results of rice type and RDV infection affecting the life-table parameters.

Parameters[a]	Rice type	*P* value	RDV infection	*P* value
r_m	MH63 vs T1C-19	0.442	MH63 vs MH63-RDV	0.819
	MH63-RDV vs T1C-19-RDV	0.841	T1C-19 vs T1C-19-RDV	0.316
R_0	MH63 vs T1C-19	0.386	MH63 vs MH63-RDV	0.068
	MH63-RDV vs T1C-19-RDV	0.158	T1C-19 vs T1C-19-RDV	0.674
T	MH63 vs T1C-19	0.892	MH63 vs MH63-RDV	0.123
	MH63-RDV vs T1C-19-RDV	0.611	T1C-19 vs T1C-19-RDV	0.637
DT	MH63 vs T1C-19	0.445	MH63 vs MH63-RDV	0.790
	MH63-RDV vs T1C-19-RDV	0.833	T1C-19 vs T1C-19-RDV	0.310
λ	MH63 vs T1C-19	0.442	MH63 vs MH63-RDV	0.822
	MH63-RDV vs T1C-19-RDV	0.842	T1C-19 vs T1C-19-RDV	0.317

Note: All life-table parameters were calculated and analyzed using an SAS program written by Maia *et al.*[39] using the jackknife method ($P < 0.05$). [a]r_m: the intrinsic rate of natural increase; R_0: the net reproductive rate; T: the mean generation time; DT: the doubling time; λ: the finite capacity of increase.
